# A Comparison of the Sensitivity and Fecal Egg Counts of the McMaster Egg Counting and Kato-Katz Thick Smear Methods for Soil-Transmitted Helminths

**DOI:** 10.1371/journal.pntd.0001201

**Published:** 2011-06-14

**Authors:** Bruno Levecke, Jerzy M. Behnke, Sitara S. R. Ajjampur, Marco Albonico, Shaali M. Ame, Johannes Charlier, Stefan M. Geiger, Nguyen T. V. Hoa, Romuald I. Kamwa Ngassam, Andrew C. Kotze, James S. McCarthy, Antonio Montresor, Maria V. Periago, Sheela Roy, Louis-Albert Tchuem Tchuenté, D. T. C. Thach, Jozef Vercruysse

**Affiliations:** 1 Department of Virology, Parasitology and Immunology, Faculty of Veterinary Medicine, Ghent University, Merelbeke, Belgium; 2 School of Biology, University of Nottingham, Nottingham, United Kingdom; 3 Department of Gastrointestinal Sciences, Christian Medical College, Vellore, India; 4 Public Health Laboratory, Ivo de Carneri, Pemba Island, Zanzibar, Tanzania; 5 Instituto René Rachou, Fundação Oswaldo Cruz, Belo Horizonte, Brazil; 6 National Institute for Malariology, Parasitology and Entomology, Hanoi, Vietnam; 7 Centre for Schistosomiasis and Parasitology, Faculty of Sciences, University of Yaoundé I, Yaoundé, Cameroon; 8 Division of Livestock Industries, Commonwealth Scientific and Industrial Research Organisation, Brisbane, Australia; 9 Queensland Institute for Medical Research, University of Queensland, Herston, Australia; 10 Department of Neglected Tropical Diseases, World Health Organization, Geneva, Switzerland; University of Kelaniya, Sri Lanka

## Abstract

**Background:**

The Kato-Katz thick smear (Kato-Katz) is the diagnostic method recommended for monitoring large-scale treatment programs implemented for the control of soil-transmitted helminths (STH) in public health, yet it is difficult to standardize. A promising alternative is the McMaster egg counting method (McMaster), commonly used in veterinary parasitology, but rarely so for the detection of STH in human stool.

**Methodology/Principal Findings:**

The Kato-Katz and McMaster methods were compared for the detection of STH in 1,543 subjects resident in five countries across Africa, Asia and South America. The consistency of the performance of both methods in different trials, the validity of the fixed multiplication factor employed in the Kato-Katz method and the accuracy of these methods for estimating ‘true’ drug efficacies were assessed. The Kato-Katz method detected significantly more *Ascaris lumbricoides* infections (88.1% *vs.* 75.6%, *p*<0.001), whereas the difference in sensitivity between the two methods was non-significant for hookworm (78.3% *vs.* 72.4%) and *Trichuris trichiura* (82.6% *vs.* 80.3%). The sensitivity of the methods varied significantly across trials and magnitude of fecal egg counts (FEC). Quantitative comparison revealed a significant correlation (Rs >0.32) in FEC between both methods, and indicated no significant difference in FEC, except for *A. lumbricoides,* where the Kato-Katz resulted in significantly higher FEC (14,197 eggs per gram of stool (EPG) *vs.* 5,982 EPG). For the Kato-Katz, the fixed multiplication factor resulted in significantly higher FEC than the multiplication factor adjusted for mass of feces examined for *A. lumbricoides* (16,538 EPG *vs.* 15,396 EPG) and *T. trichiura* (1,490 EPG *vs.* 1,363 EPG), but not for hookworm. The McMaster provided more accurate efficacy results (absolute difference to ‘true’ drug efficacy: 1.7% *vs.* 4.5%).

**Conclusions/Significance:**

The McMaster is an alternative method for monitoring large-scale treatment programs. It is a robust (accurate multiplication factor) and accurate (reliable efficacy results) method, which can be easily standardized.

## Author Summary

Currently, in public health, the reduction in the number of eggs excreted in stools after drug administration is used to monitor the efficacy of drugs against parasitic worms. Yet, studies comparing diagnostic methods for the enumeration of eggs in stool are few. We compared the Kato-Katz thick smear (Kato-Katz) and McMaster egg counting (McMaster) methods, which are commonly used diagnostic methods in public and animal health, respectively, for the diagnosis and enumeration of eggs of roundworms, whipworms and hookworms in 1,536 stool samples from children in five trials across Africa, Asia and South America. The Kato-Katz method was the most sensitive for the detection of roundworms, but there was no significant difference in sensitivity between the methods for hookworms and whipworms. The sensitivity of the methods differed across the trials and magnitude of egg counts. The Kato-Katz method resulted in significantly higher egg counts, but these were subject to lack of accuracy caused by intrinsic properties of this method. McMaster provided more reliable estimates of drug efficacies. We conclude that the McMaster is an alternative method for monitoring large-scale treatment programs. It allows accurate monitoring of drug efficacy and can be easily performed under field conditions.

## Introduction

Infection with soil-transmitted helminths (STH), including *Ascaris lumbricoides, Trichuris trichiura* and hookworm (*Ancylostoma duodenale* and *Necator americanus*) are of major importance for public health in tropical and subtropical countries [1], [2]. Current approaches proposed for controlling STH infections entail periodic large-scale administration of anthelmintic drugs, particularly targeting school-aged children [3], [4]. Since such large-scale interventions are likely to intensify as more attention is given to these neglected tropical diseases [5], monitoring drug efficacy will assume increasing importance for assessment of drug efficacy [6] and for detection of the emergence of resistance [7], [8].

A weakness of published studies reporting anthelmintic efficacy in human trials has been the focus on qualitative diagnosis of infections (presence/absence of STH eggs in stool) after treatment, that is, on the cure rate. Quantitative studies, reporting the reductions in the number of eggs excreted are published more rarely (fecal egg count reduction (FECR)) [9], yet are likely to provide the best summary measure for assessment of anthelmintic efficacy in large-scale treatment programs [10]. Although this implies the need for methods to accurately quantify egg excretion levels, studies where more than one coprological method based on fecal egg counts (FEC) has been used, are scarce. In addition, little is known about the variability in qualitative and quantitative diagnosis by these methods between different laboratories [11] or about the accuracy of the methods for estimating drug efficacies in monitoring programs.

To date, the Kato-Katz thick smear method (Kato-Katz) is the diagnostic method recommended by the World Health Organization (WHO) for the quantification of STH eggs in human stool [12], because of its simple format and ease-of-use in the field. The chief limitation of the Kato-Katz method, however, arises when it is used with the objective of simultaneous assessment of STH in fecal samples from subjects with multiple species infections. This is because helminth eggs of different species of helminths appear at different time intervals (clearing times). In addition, hookworm eggs rapidly disappear in cleared slides, resulting in false negative test results if the interval between preparation and examination of the slides is too long (>30 min). These properties have impeded standardization of the Kato-Katz method in large-scale studies at different study sites [13]–[15]. Moreover, quantification of the intensity of egg excretion is based on a fixed volume of feces, rather than the mass of feces examined. Its quantitative performance is, therefore, questionable, as the intensity of eggs excreted is expressed as the number of eggs per gram of stool (EPG) [16], and the density of feces can vary. This potential bias in the value of FEC is likely to be important in programs monitoring drug efficacy by the Kato-Katz, where it may introduce additional variation in the results of FECR and broaden the confidence levels of the resulting statistical parameters.

A recent study in non-human primates, demonstrated that the McMaster egg counting method (McMaster) holds promise for the assessment of the efficacy of anthelmintics by FECR [17], as it provided accurate estimates of FEC, and was very easy to use, making it particularly suitable for use in poorly equipped and often short-staffed laboratories. However, despite the fact that McMaster is the method of choice for efficacy monitoring programs in veterinary medicine [18], its performance for the detection and enumeration of STH eggs in human public health remains unknown.

Therefore, a multinational study was conducted to evaluate the relative performance of the McMaster and Kato-Katz methods for monitoring drug efficacy in STH in humans. To this end, these methods were compared for both qualitative and quantitative detection of STH in human populations in Brazil, Cameroon, India, Tanzania and Vietnam. The three specific objectives of the current study were (i) to assess the consistency of the performance of these two methods in trials conducted in these different countries located in three continents; (ii) to validate the fixed multiplication factor employed in the Kato-Katz method; and (iii) to assess the accuracy of both methods for estimating drug efficacies based on FECR.

## Methods

### Ethics statement

The overall protocol of the study was approved by the ethics committee of the Faculty of Medicine, Ghent University (Nr B67020084254), followed by a separate local ethical approval for each study site. For Brazil, approval was obtained from the institutional review board from Centro de Pesquisas René Rachou (Nr 21/2008), for Cameroon from the national ethics committee (Nr 072/CNE/DNM08), for India from the institutional review board of the Christian Medical College (Nr 6541), for Tanzania (Nr 20) from the Zanzibar Health Research Council and the Ministry of Health and Social Welfare, for Vietnam by the Ministry of Health of Vietnam. All subjects included in the study, or the parents in the case of school-aged children, signed an informed consent form. The clinical trial in this study was registered under the ClinicalTrials.gov identifier NCT01087099.

### Study sites and population

The study was undertaken in five countries across Africa (Cameroon, Tanzania), Asia (India, Vietnam) and South America (Brazil). For Brazil, Cameroon, Tanzania, and Vietnam, the subjects involved also participated in a multinational trial of the efficacy of a single-dose albendazole (400 mg) against STH infections, which has been presented elsewhere [10]. It is important to note that here we do not make comparison between countries as such, but rather between five distinct trials conducted in five countries in geographically contrasting regions of the world, and reference to country is only for the purpose of distinguishing between specific trials. For this multinational efficacy trial, only subjects meeting the required criteria were included: attending school, aged of 4–18 years, not experiencing a severe concurrent medical condition or diarrhea at time of first sampling. For the trial conducted in India, stool samples of patients presented at the Christian Medical College hospital in August 2009 were included. A subset of at least 100 subjects (first screened) from each site was included in the analysis. This sample size was based on available prevalence data [19]–[23], and was sufficient in size to enable analysis by logistic regression modeling (10 infected subjects per predictor included in the model) [24].

### Parasitological methods

All stool samples were processed by the McMaster and the Kato-Katz methods as described below. For each stool sample, both methods were applied on the same day by experienced laboratory technicians blinded to any preceding test results.

#### McMaster

The McMaster method was based on the modified McMaster described by the Ministry of Agriculture, Fisheries, and Food (1986) [25]. Two grams of stool were suspended in 30 ml of saturated salt solution at room temperature (density ∼1.2, prepared by adding NaCl to 5 l of warm distilled water (40–50°C) until no more salt went into solution and the excess settled on the bottom of the container). The fecal suspension was poured three times through a wire mesh (aperture of 250 µm) to remove large debris. Then, 0.5 ml aliquots were added to each of the two chambers of a McMaster slide (http://www.mcmaster.co.za). Both chambers were examined under a light microscope using a 100x magnification and the FEC, expressed as EPG for each helminth species, were obtained by multiplying the total number of eggs by 50. A tutorial for performing the McMaster is made available at http://www.vetparasitology.ugent.be/page30/page30.html.

#### Kato-Katz

The Kato-Katz thick smears were prepared as described by WHO (1991) [12] on microscope slides using a square template with a hole diameter of 6 mm and depth of 1.5 mm, which is assumed to sample 41.7 mg of feces. All samples were examined within 30–60 min for the presence of hookworm and re-examined after ∼2 hours for the remaining STH eggs. The number of helminth eggs was counted on a per species basis and multiplied by 24 to obtain the FEC in units of EPG. In addition, in Tanzania and Cameroon, the validity of the multiplication factor was investigated by weighing the mass of feces examined, and then by comparing FEC based on the fixed multiplication factor of 24 with those based on a multiplication factor adjusted for the actual weight of the amount of feces examined. To this end, microscope slides were weighed (scale precision of 0.01 g) individually, without and with their aliquot of stool. The multiplication factor adjusted for the mass of feces examined was therefore 1 over the mass of the feces examined in grams (mass slide with feces – mass slide without feces).

### Statistical analysis

**Figure 1:**
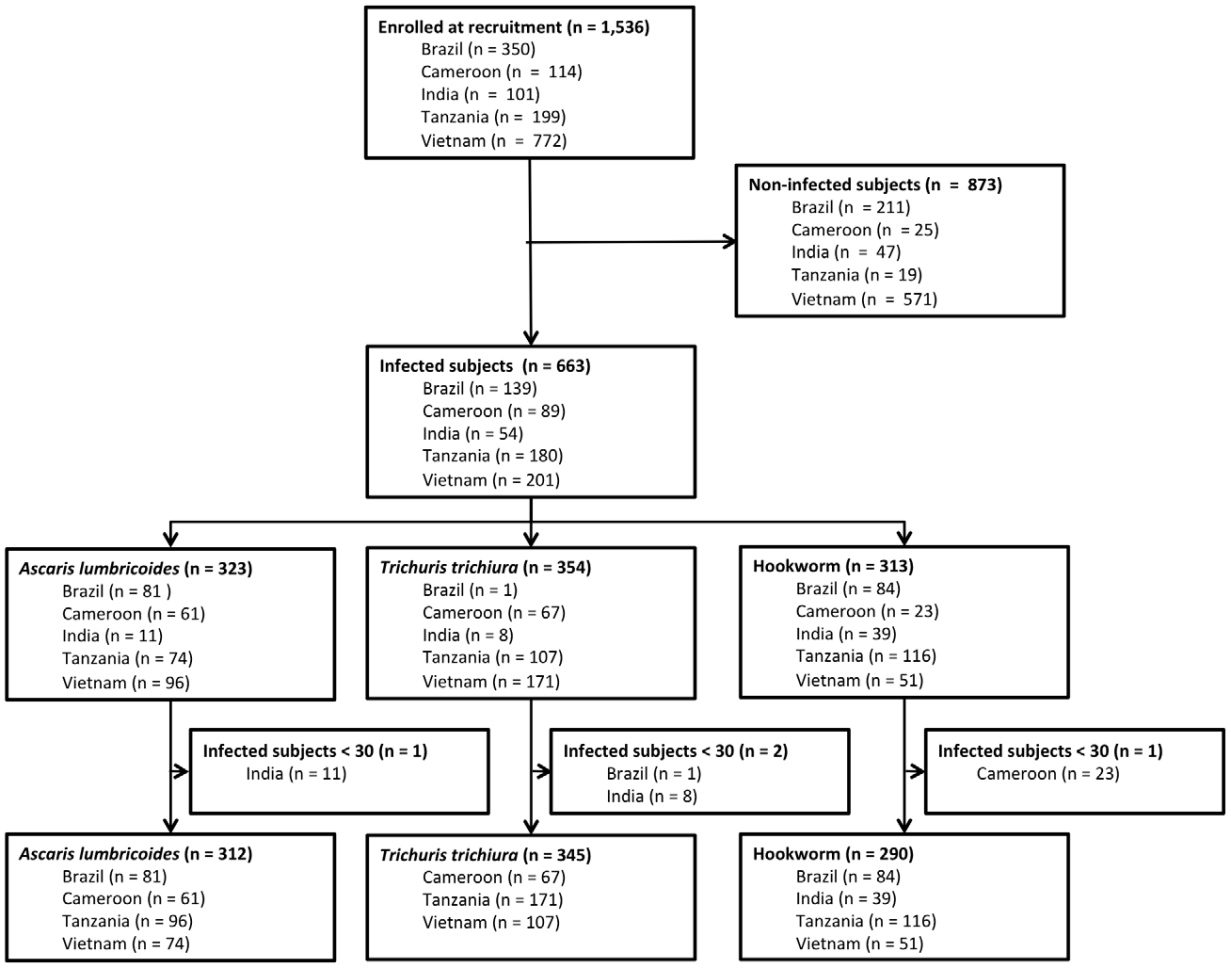
The number of subjects involved in the statistical analysis for agreement in test results.

As described below both diagnostic methods were compared qualitatively (sensitivity and negative predictive value (NPV)) and quantitatively (FEC) for each of the three STH species. In addition, the validity of the fixed multiplication factor for the Kato-Katz was examined. Finally, the accuracy of both methods for estimating drug efficacy by means of FECR was assessed. Both the qualitative and quantitative comparisons for each of the three STH separately were based only on subjects meeting the following inclusion criteria: (i) excreting STH eggs and (ii) originating from a trial were a minimal of 30 infected subjects were detected at the initial survey. The number of subjects enrolled, the occurrence of STH and the number of subjects included for this qualitative and quantitative comparison are shown in Figure 1.

#### Qualitative agreement

Sensitivity was calculated for each method, using the combined results of both methods as the diagnostic ‘gold’ standard. Therefore, the specificity of both methods was set at 100%, as indicated by the morphology of the eggs. Differences in sensitivity between methods was assessed by the Z-test. The variation in sensitivity within each method was explored by a logistic regression model, which was fitted for each of the two methods with their test result (positive/negative) as the outcome, the mean FEC of both methods as covariate, and trial as a factor (five levels: Brazil, Cameroon, India, Tanzania, and Vietnam). The final models were evaluated from the full factorial model (including interactions) by a backward selection procedure (least significant predictor was step wise omitted from the model) using the *χ* ^2^ likelihood ratio statistic. The level of significance was set at *p* <0.05. The predictive power of the final models was evaluated by the proportion of the observed outcome that was correctly predicted by the model. To this end, an individual probability >0.5 was set as a positive test result, and negative if different. Finally, the sensitivity for each of the observed values of the covariate and factor, was calculated based on these models (R Foundation for Statistical Computing, version 2.10.0). The NPV was calculated according the theorem of Bayes. The 95% confidence intervals (CIs) for NPV were obtained by statistical simulation (R Foundation for Statistical Computing, version 2.10.0).
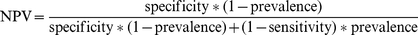



#### Quantitative agreement

The agreement in quantitative test results was estimated by the Spearman rank correlation coefficient (Rs) (SAS 9.1.3, SAS Institute Inc.; Cary, NC, USA). The Wilcoxon signed rank test was used to test for differences in FEC between the methods. Furthermore, samples were subdivided into low, moderate, and high egg excretion intensities according to thresholds proposed by WHO [9]; for *A. lumbricoides* these were 1–4,999 EPG, 5,000–49,999 EPG, and >49,999 EPG; for *T. trichiura* these were 1–999 EPG, 1000–9,999 EPG, and >9,999 EPG; and for hookworm these were 1–1,999 EPG, 2,000–3,999 EPG, and >3,999 EPG, respectively. Finally, the agreement in the assignment to these three levels of egg excretion intensity by the McMaster and Kato-Katz methods was evaluated by the Cohen’s kappa statistic (κ). The value of this statistic indicates a slight (κ<0.2), fair (0.2≤κ<0.4), moderate (0.4≤κ<0.6), substantial (0.6≤κ<0.8) and an almost perfect agreement (κ ≥ 0.8) (R Foundation for Statistical Computing, version 2.10.0).

#### Validity of the multiplication factor of Kato-Katz

The validity of the fixed multiplication factor used in the Kato-Katz method was evaluated using three approaches. First, the accuracy and precision of this multiplication factor were assessed by the mean and 95% CI of the multiplication factor adjusted for the mass of feces actually examined. Second, differences in the multiplication factor adjusted for feces between the trials conducted in Tanzania and Cameroon were assessed by the Mann-Witney U test. Finally, the quantitative agreement between Kato-Katz tests with the fixed and the adjusted multiplication factors was re-analyzed as described above in the section “Quantitative agreement”.

#### The accuracy of estimating drug efficacy

Statistical simulations were conducted to assess the ability of the McMaster and Kato-Katz methods to estimate the reduction in FEC after chemotherapy under varying drug efficacies and baseline FEC. These simulations focused on *T. trichiura*, since this STH requires a more intensive treatment regime for clearance [10], [26], [27] and there is already presumptive evidence that this parasite poses a higher risk for the development of drug resistance [28]. Therefore, we used observed data from the trials conducted in Cameroon, Tanzania, and Vietnam (Figure 1), in particular the sensitivities derived from these trials for *T. trichiura* for each of the two methods across the range of observed intensities of infection. Drug efficacies of 90%, 95% and 99% were fitted virtually into the simulation model for each of the two methods with pre-drug administration FEC (pre-DA FEC) of 100, 250, 500, 750 and 1,000 EPG. The values for the pre-DA FEC were based on FEC *T. trichiura* previously reported in efficacy trials [10], [26]. To fully understand this experiment, the various steps will be illustrated in the following example: First, the study population before the administration of drugs was defined. In this example, we included 100 subjects each excreting 1,000 EPG ( =  ‘true’ pre-DA FEC). However, the actual number of subjects diagnosed largely depends on the sensitivity of the method used. Therefore, the number of subjects diagnosed equaled the product of the number of subjects infected (*in casu* 100) and the sensitivity of the method (the sensitivity values being those actually recorded in the three trials in Cameroon, Tanzania, and Vietnam, but in this example only that from Cameroon) (Table S1). Because pre-DA FECs were high in this example, we assumed that both methods were able to diagnose almost all infected subjects (sensitivity_McMaster_ ∼99%; sensitivity_Kato-Katz_ ∼93%). The ‘observed’ pre-DA FEC was set at the nearest multiplicity of 50 for the McMaster (i.e., 50×20 = 1,000 EPG) and 24 for the Kato-Katz (i.e., 24×42 = 1,008 EPG). To obtain a ‘true’ drug efficacy (TDE) of 99%, the ‘true’ pre-DA FEC were multiplied by 1% (i.e., 100% - TDE), resulting in a ‘true’ FEC after drug administration ( =  ‘true’ post-DA FEC) of 10 EPG. However, the number of infected subjects diagnosed will again depend on the sensitivity of the method and this will change from pre-DA FEC because FEC have dropped and are now much lower (data from the trials revealed that sensitivity was lower when FEC were low). For this example, for an EPG of 10, we use a sensitivity of approximately 12.0% for the McMaster and a sensitivity of approximately 77% for the Kato-Katz, based on actual sensitivities recorded for this intensity of infection with *T. trichiura* in the trial in Cameroon. Subsequently, the FEC were determined as described above, resulting in an ‘observed’ post-DA FEC of 50 and 24, respectively. Finally, the ‘observed’ drug efficacy (ODE) was calculated using the formula below, revealing an ODE of 99.4% for McMaster and 98.2% for Kato-Katz, for a pre-DA FEC of 1,000 EPG, a known TDE of 99%, and sensitivity values for *T. trichiura* from the trial in Cameroon. 
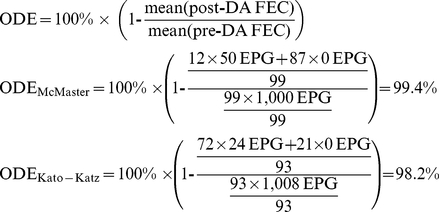



In total 90 simulations were performed ( = 2 (diagnostic methods) ×3 (trials) ×3 (TDE) ×5 pre-DA FEC). For each simulation, the absolute difference between the TDE and the ODE ( =  bias) was calculated. Finally, the Wilcoxon signed rank test was used to test for differences in bias between the diagnostic methods.

## Results

### The agreement in qualitative test results

The prevalence and the agreement in qualitative test results (sensitivity and NPV) between Kato-Katz and McMaster are summarized in Table 1. Overall, each of the three STH showed similar prevalence, ranging from 20.3% for hookworm over 21.7% for *A. lumbricoides* to 26.1% for *T. trichiura*. The Kato-Katz method (88.1%) was more sensitive for the detection of *A. lumbricoides* infections compared to the McMaster method (75.6%) (z = 4.01, *p*<0.001, n = 312). For hookworm (78.3% *vs.* 72.4%) and *T. trichiura* (82.6% *vs.* 80.3%), the difference was non-significant resulting in a *p*-value of 0.10 (z = 1.65, n = 290) and 0.43 (z = 0.78, n = 345), respectively. The NPV for both methods was higher than 93% for all three STH. There was a large overlap in 95% CI between the two methods, except for *A. lumbricoides* where there was no overlap in 95% CI.

**Table 1:** The qualitative agreement between McMaster and Kato-Katz for the detection of soil-transmitted helminths.

	Study site	Prevalence (%)	Sensitivity (%)			NPV (%) (95% CI)	
			McMaster	Kato-Katz	*p*-value	McMaster	Kato-Katz
***A. lumbricoides***		**21.7**	**75.6**	**88.1**	**<0.001**	**93.6 (92.2–95.0)**	**96.8 (95.7–97.8)**
	Brazil	23.1	67.9	100	<0.001	91.2 (87.8–94.2)	100
	Cameroon	53.5	85.2	90.2	0.40	85.5 (76.0–93.6)	89.9 (81.6–96.7)
	Tanzania	37.1	81.3	92.7	0.04	85.1 (78.2–91.1)	93.7 (88.8–97.8)
	Vietnam	12.3	68.9	67.6	0.85	96.8 (95.4–98.0)	96.7 (95.3–97.9)
***T. trichiura***		**26.1**	**80.3**	**82.6**	**0.43**	**93.5 (91.9–94.9)**	**94.2 (92.7–95.6)**
	Cameroon	58.8	83.6	97.0	0.01	81.1 (70.4–90.5)	95.9 (89.4–100)
	Tanzania	53.8	91.2	90.6	0.88	65.2 (50.2–79.1)	63.5 (48.9–77.3)
	Vietnam	22.1	60.7	60.7	1.00	94.0 (92.2–95.7)	94.0 (92.2–95.7)
**Hookworm**		**20.3**	**72.4**	**78.3**	**0.10**	**93.4 (92.0–94.7)**	**94.7 (93.4–95.9)**
	Brazil	24.0	71.4	95.2	<0.001	91.7 (88.3–94.7)	98.5 (96.9–99.7)
	India	38.6	76.9	92.3	0.05	87.3 (78.6–94.4)	95.3 (89.4–100)
	Tanzania	58.3	74.1	73.3	0.89	73.4 (64.7–81.1)	72.9 (64.4–80.9)
	Vietnam	6.6	66.7	51.0	0.10	97.7 (96.5–98.7)	96.7 (95.3–97.9)

There was considerable variation between the different trials (countries) in prevalence, sensitivity and to a lesser extent in NPV. *A. lumbricoides* was the most prevalent species in Cameroon (53.5%), but eggs of this parasite were rarely detected in the Vietnamese trial (12.3%). *T. trichiura* (53.8%) and hookworm (58.3%) were the most prevalent STHs in Tanzania, whereas in Vietnam they were less prevalent (22.1%) and even relatively rare (6.6%), respectively. The explanation for the significant differences in prevalence was beyond the scope of the present study.

The sensitivity of the McMaster method varied from 67.9% (Brazil) to 85.2% (Cameroon) for *A. lumbricoides*, from 60.7% (Vietnam) to 91.2% (Tanzania) for *T. trichiura,* and from 66.7% (Cameroon) to 76.9% (India) for hookworm. The sensitivity of the Kato-Katz method ranged from 67.6% (Vietnam) to 100% (Brazil), from 60.7% (Vietnam) to 97.0% (Cameroon), and from 51.0% (Vietnam) to 95.2% (Brazil) for *A. lumbricoides*, *T. trichiura* and hookworm, respectively. In Brazil (100% *vs.* 67.9%, n = 81, z = 6.09, *p*<0.001) and Tanzania (92.7% *vs.* 81.3%, n = 74, z = 2.09, *p* = 0.04), significantly more *A. lumbricoides* infections were diagnosed with Kato-Katz, than with McMaster. For *T. trichiura*, this was the case in Cameroon (97.0% *vs.* 83.6%, n = 67, z = 2.69, *p* = 0.01). For hookworm, a significant difference in sensitivity between the two methods was found only in Brazil (95.2% *vs.* 71.4%, n = 84, z = 4.4, *p*<0.001).

**Figure 2:**
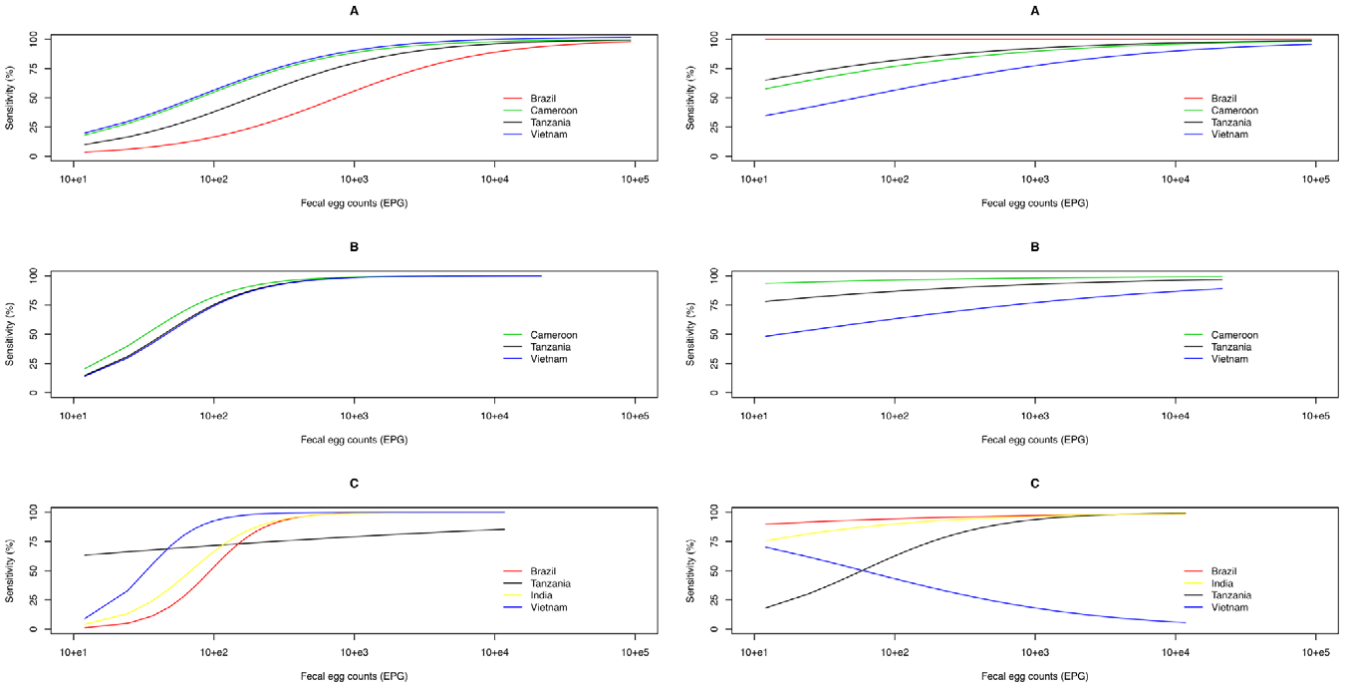
The sensitivity for McMaster and Kato-Katz. The predicted sensitivity derived from logistic regression for McMaster (left graphs) and Kato-Katz (right graphs) for *A. lumbricoides* (A), *T. trichiura* (B), and hookworm (C) in the different trials (countries) involved.

This variation in sensitivity of both methods could be largely explained by the magnitude of the FEC and ‘trials’ (more than 80% of the outcome was correctly predicted). The predicted sensitivity of the McMaster and Kato-Katz methods for the detection of STH in the different trials is illustrated by Figure 2. For the McMaster method, the sensitivity was equally affected by FEC at all trials for *A. lumbricoides* (χ^2^_1_ = 112.6, *p*<0.001) and *T. trichiura* (χ^2^_1_ = 78.0, *p*<0.001), but not for hookworm (χ^2^_1_ = 1.0, *p* = 0.31), where the effect of FEC on the sensitivity differed between the different trials (lines for trials in different countries cross one another) (two-way interaction FEC x trial, χ^2^_3_ = 36.9, *p*<0.001). A significant difference between trials was found for *A. lumbricoides* (χ^2^_3_ = 17.3, *p*<0.001) and hookworm (χ^2^_3_ = 33.5, *p*<0.001), but not for *T. trichiura* (lines close to one another and overlapping) (χ^2^_2_ = 0.9, *p* = 0.64). Analysis of the Kato-Katz method yielded similar models, but they differed from the results of the analysis of the McMaster method in four ways. First, the effect of intensity of FEC was less pronounced (flat curves for *A. lumbricoides* and *T. trichiura*: χ^2^_1_ = 22.4, *p*<0.001 and χ^2^_1_ = 3.9, *p*<0.05, respectively). Second, high FEC contributed significantly to the ability of Kato-Katz to detect hookworm (χ^2^_1_ = 22.0, *p*<0.001). Third, significant differences between trials occurred with *T. trichiura* (χ^2^_2_ = 27.8, *p*<0.001). Finally, a drop in sensitivity was observed at high FEC in the trial in Vietnam for hookworm (χ^2^_3_ = 16.4, *p*<0.001).

**Figure 3:**
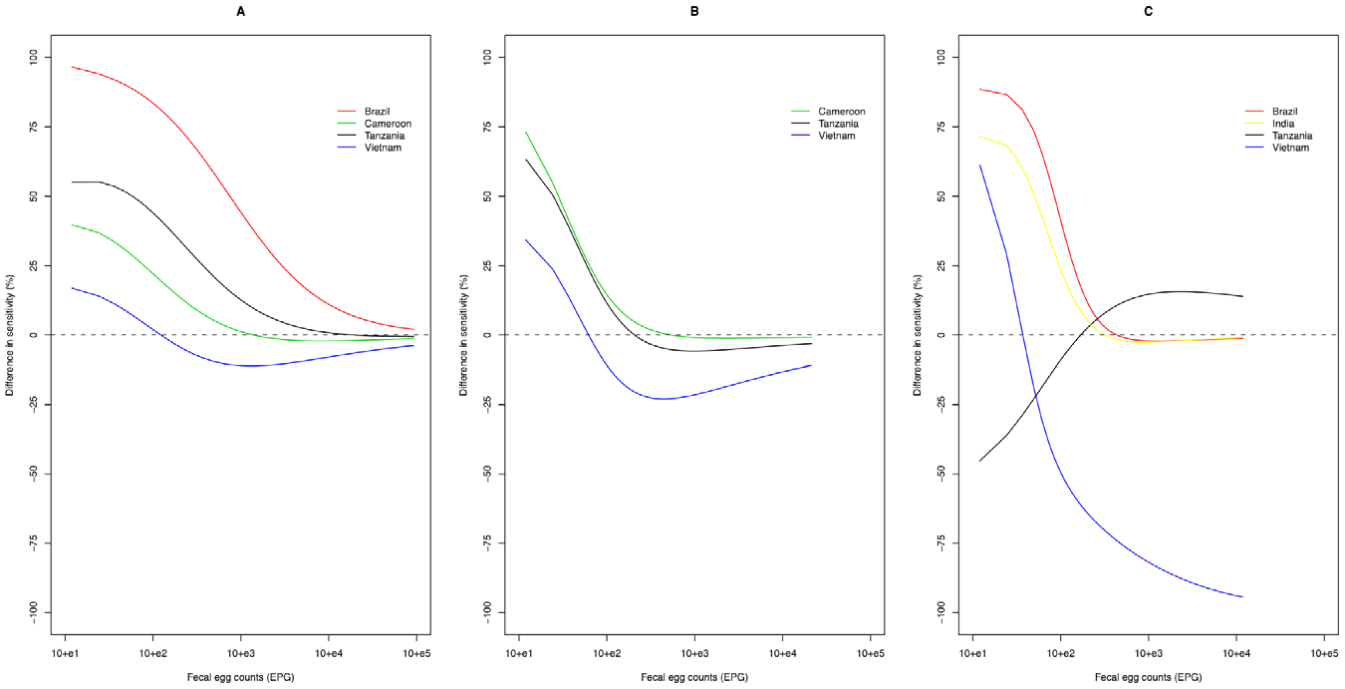
Differences in sensitivity between Kato-Katz and McMaster. Differences in sensitivity (Sensitivity_Kato-Katz_-Sensitivity_McMaster_) for *A. lumbricoides* (A), *T. trichiura* (B), and hookworm (C) in the different trials (countries) involved.

Figure 3 shows the differences in predicted sensitivity between the two methods. Overall, the McMaster method often failed to detect infection when the intensity of egg excretion was low, but performed at least as well as Kato-Katz as the FEC increased. This decrease in differences in sensitivity across increasing FEC was also found more or less for each of the three STH in all trials. An exception was Vietnam, where the McMaster method was more sensitive compared to Kato-Katz as FEC increased.

The NPV of both methods was high (>80%) for each of the three STH in all trials (Table 1), except for the detection of *T. trichiura* (McMaster: 65.2%; Kato-Katz: 63.5%) and hookworm (McMaster: 73.4%; Kato-Katz: 72.9%) in Tanzania. In the majority of the cases, there was a large overlap in the 95% CI of both diagnostic methods, except in the Brazilian trial for the detection of *A. lumbricoides* (McMaster: [87.8–94.2%] *vs.* Kato-Katz: [100–100%]) and hookworm ([88.3–94.7%] *vs.* [96.9–99.7%]) and in Cameroon for *T. trichiura* ([70.4–90.5%] *vs.* [89.4–100%]). In each of these trials, the overlap was either small or absent.

### Agreement in quantitative test results

Overall there was a significant correlation between the FEC of the McMaster and those obtained by Kato-Katz (*A. lumbricoides*: Rs  = 0.70, n = 312, *p*<0.001; *T. trichiura*: Rs * = *0.49, n = 345, *p*<0.001; hookworm: Rs  = 0.32, n = 290, *p*<0.001) (Table 2). Assessment of egg excretion intensity by the Kato-Katz resulted in significantly more eggs of *A. lumbricoides* (14,197 EPG *vs.* 5,982, n = 312, *p*<0.001), but not for hookworm (468 EPG *vs.* 409, n = 290, *p* = 0.10) and *T. trichiura* (784 EPG *vs.* 604, n = 345, *p* = 1.00). However, these findings were not consistent across the different trials. A significant positive correlation between both methods was found for each of the three STH in all countries (Rs  = 0.28–0.88, *p*<0.05), except for trials in Tanzania and Vietnam. In Tanzania, no significant correlation was found between the two methods for the quantification of hookworm eggs (R_s_ = −0.05, n = 116, *p* = 0.56), while in the trial in Vietnam, a significant negative correlation was found for *T. trichiura* (Rs  = −0.24, n = 107, *p* = 0.01) and hookworm (R_s_  = −0.49, n = 51, *p*<0.001). A significant difference in the enumeration of STH eggs between the Kato-Katz and McMaster methods was found for Brazil, Cameroon, and Vietnam. In both the Brazilian and Cameroonian trials, the Kato-Katz method yielded higher FEC compared to the McMaster method. In the Vietnamese trial, the McMaster method resulted in detection of more *T. trichiura* and hookworm eggs. In trials in India and Tanzania, no significant differences between the methods were found.

**Table 2:** The quantitative agreement in fecal egg counts (FEC) between McMaster and Kato-Katz.

Country		n	Mean FEC (EPG)		Rs (*p*-value)	*p-* value for Δ _FEC_
			McMaster	Kato-Katz		
***A. lumbricoides***		**312**	**5,982**	**14,197**	**0.70 (<0.001)**	**<0.001**
	Brazil	81	6,490	25,079	0.88 (<0.001)	<0.001
	Cameroon	61	10,643	2,0531	0.82 (<0.001)	<0.001
	Tanzania	74	4,460	6,876	0.58 (<0.001)	0.08
	Vietnam	96	3,559	6,560	0.28 (0.015)	0.20
***T. trichiura***		**345**	**604**	**784**	**0.49 (<0.001)**	**1.00**
	Cameroon	67	1,168	1,938	0.76 (<0.001)	0.001
	Tanzania	171	671	769	0.38 (<0.001)	0.60
	Vietnam	107	143	84	−0.24 (0.01)	0.006
**Hookworm**		**290**	**409**	**468**	**0.32 (<0.001)**	**0.10**
	Brazil	84	422	796	0.66 (<0.001)	<0.001
	India	39	1,031	1,630	0.67 (<0.001)	0.57
	Tanzania	116	300	783	−0.05 (0.56)	0.09
	Vietnam	51	162	32	−0.49 (<0.001)	<0.001

Rs: Spearman correlation coefficient; Δ_FEC_: FEC_Kato-Katz_ – FEC_McMaster_.

**Figure 4:**
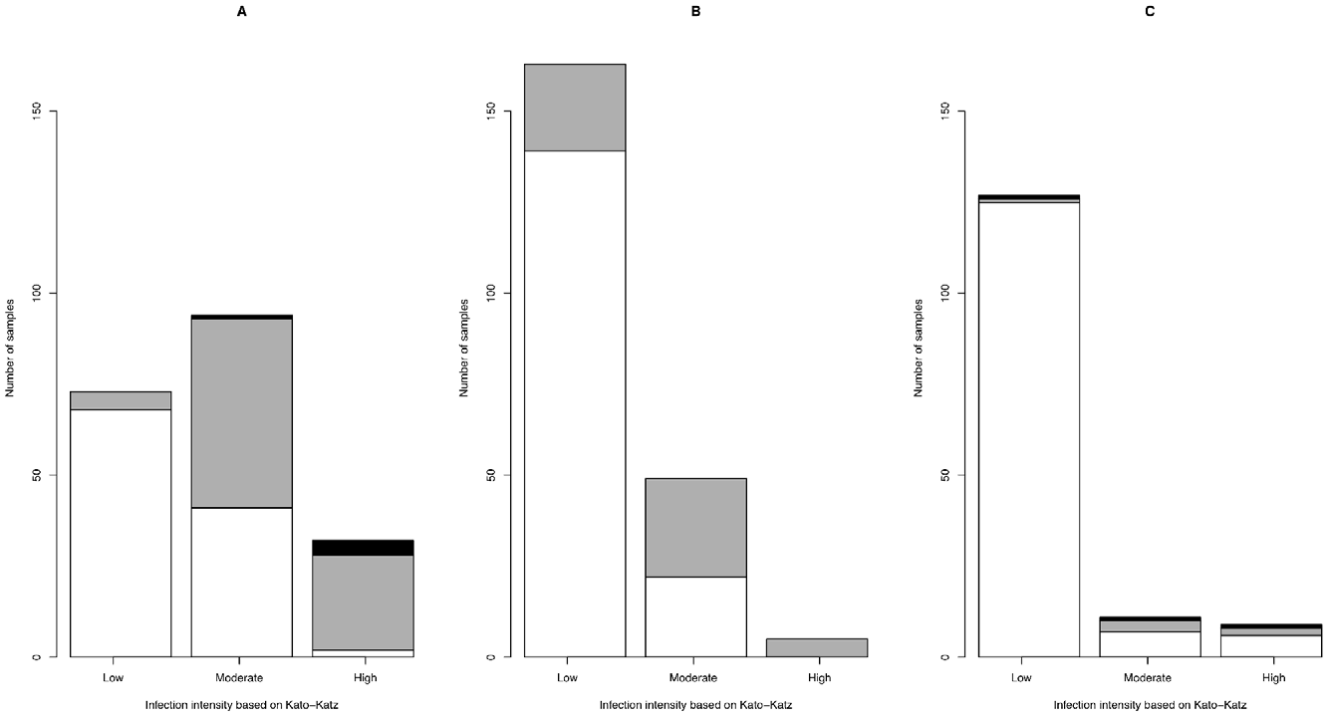
The agreement in the assignment to egg excretion intensity obtained by McMaster and Kato-Katz. The distribution of egg excretion intensity obtained by the McMaster method (low [white], moderate [grey], and high [black] over the egg excretion intensity observed by the Kato-Katz method for *A. lumbricoides* (A) (n = 199), *T. trichiura* (B) (n = 217), and hookworm (C) (n = 147).

Overall, there was a fair agreement (0.2≤κ<0.4) between the methods in the assignment of the samples to the three levels of egg excretion intensity as recommended by WHO (*A. lumbricoides*: κ = 0.37 (n = 199, *p*<0.001); *T. trichiura*: κ = 0.39 (n = 217, *p*<0.001); hookworm: κ = 0.34 (n = 147, *p*<0.001). As shown in the Figure 4, the McMaster method often assigned the samples to a lower level of egg excretion intensity compared to the Kato-Katz method.

### The validity of the multiplication factor employed in the Kato-Katz

The mass of feces was measured in 207 Kato-Katz thick smears (Cameroon, n = 107; Tanzania, n = 100) in order to assess the validity of the multiplication factor used. Overall, the adjusted multiplication factor was 23.7, but it was subject to considerable variation (95% CI: [14.3–66.7]). This variation was observed in both trials (Cameroon 23.3 [13.4–83.3], and Tanzania 23.7 [15.3–54.3]) (*p* = 0.82). Table 3 summarizes the quantitative agreement between the FEC based on the fixed and adjusted multiplication factor, respectively. There was a high correlation between both approaches (R_s_  = 0.98, n = 39–146, *p*<0.001), regardless of in which country the trial was based. However, FEC obtained on the fixed multiplication factor were significantly higher compared to those adjusted for the mass of feces examined for *A. lumbricoides* (16,538 EPG *vs.* 15,396 EPG, n = 99, *p*<0.001), *T. trichiura* (1,490 EPG *vs.* 1,363 EPG, n = 146, *p*<0.001), but not for hookworm (351 EPG *vs.* 301 EPG, n = 39, *p* = 0.05). These findings were confirmed in both countries, though not significant in the case of *A. lumbricoides* in Tanzania. Despite the differences in FEC, there was a substantial to almost perfect agreement in the assignment to the different levels of egg excretion intensity between both approaches (κ*_A. lumbricoides_*  = 0.93, n = 99, *p*<0.001; κ*_T. trichiura_*  = 0.89, n = 146, *p*<0.001; κ_hookworm_  = 0.93, n = 39, *p*<0.001).

**Table 3:** The quantitative agreement in fecal egg counts (FEC) between Kato-Katz using different multiplication factors.

		n	Mean FEC (EPG)		Rs (*p*-value)	*p-* value for Δ _FEC_
			Fixed	Adjusted		
***A. lumbricoides***		**99**	**16,538**	**15,396**	**0.98 (<0.001)**	**<0.001**
	Cameroon	54	12,307	11,702	0.98 (<0.001)	<0.001
	Tanzania	45	4,527	3,953	0.98 (<0.001)	0.11
***T. trichuria***		**146**	**1,490**	**1,363**	**0.98 (<0.001)**	**<0.001**
	Cameroon	62	2,268	2,023	0.98 (<0.001)	0.001
	Tanzania	84	904	865	0.98 (<0.001)	0.02
**Hookworm**		**39**	**351**	**301**	**0.98 (<0.001)**	**0.05**
	Tanzania	39	351	301	0.98 (<0.001)	0.05

Rs: Spearman correlation coefficient; Δ_FEC_: FEC_fixed_ – FEC_adjusted_.

### Accuracy of estimating drug efficacy

**Figure 5:**
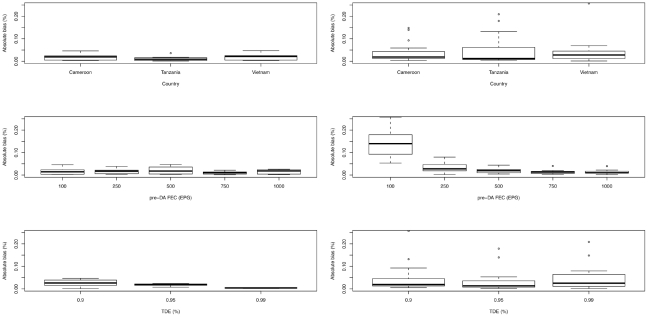
The absolute bias for McMaster and Kato-Katz in the assessment of drug efficacy. The bias (i.e., absolute value of the differences between the ‘true’ drug efficacy (TDE) and the observed fecal egg count reduction) for McMaster and Kato-Katz across the different trials (countries), pre-drug administration fecal egg counts (pre-DA FEC) and ‘true’ drug efficacies (TDE) based on predictions from statistical models.

Overall, the mean bias (departure from the TDE in either direction) was 1.7% for McMaster and 4.5% for Kato-Katz. The bias for each of the two methods by trials (different countries), by pre-DA FEC and by TDE are illustrated in Figure 5. The bias for McMaster did not exceed 5%. Differences in bias across trials were small (Cameroon: 0.3–4.6%; Tanzania: 0.1–3.6%; Vietnam: 0.3–4.7%), but there was a decrease in bias across both pre-DA FEC (100 EPG: 0.3–4.6%; 250 EPG: 0.3–3.8%; 500 EPG: 0.2–4.7%; 750 EPG: 0.1–2.1%; 1,000 EPG: 0.1–2.6%) and TDE (90%: 0.1–4.7%; 95%: 0.7–2.4%; 99%: 0.1–0.5%). The bias for Kato-Katz ranged from 0.01% to 25.7%, and decreased when pre-DA FEC increased (100 EPG: 5.3–25.7%; 250 EPG: 0.2–8.0%; 500 EPG: 0.5–4.4%; 750 EPG: 0.3–4.0%; 1,000 EPG: 0.1–4.0%). Across trials (Cameroon: 0.3–14.8%; Tanzania: 0.4–20.9%; Vietnam: 0.1–25.7%) and TDE (90%: 0.5–25.7%; 95%: 0.2–17.9%; 99%: 0.1–20.9%), the bias remained largely unchanged. McMaster was significantly more accurate in estimating FECR compared to Kato-Katz (*p* = 0.006). Yet, these differences in accuracy of FECR between the methods became non-significant when only pre-DA FEC above 100 EPG were considered (*p* = 0.40, McMaster: 1.6% (range: 0.01–4.7%), Kato-Katz: 2.0% (range: 0.01–8.0%)). A detailed overview of the calculations made is available in Table S1.

## Discussion

In the present study, the McMaster and Kato-Katz were compared for both qualitative and quantitative detection of STH infections in human populations on a scale that is unprecedented in the literature. Moreover, we assessed (i) the consistency of the performance of these two methods across five trials in different countries, (ii) the validity of a fixed multiplication factor for the Kato-Katz, and (iii) the ability of both methods to estimate a ‘true’ drug efficacy.

The qualitative comparison revealed that Kato-Katz was more sensitive for the detection of *A. lumbricoides*, but not for hookworm and *T. trichiura.* These differences in sensitivity can be explained to some extent by the intrinsic properties of the methods. In the Kato-Katz method, a larger quantity of stool is examined (Kato-Katz: 41.7 mg, McMaster: 20 mg). Moreover, this quantity of stool is determined after the larger items in fecal debris have been removed by sieving, whereas the initial quantity of stool used in the McMaster method includes large items of debris. Finally, the McMaster method is based on the flotation of eggs, but it is clear that the buoyancy of eggs differs between the different STHs. For example, it was noticed that unfertilized eggs of *A. lumbricoides* (heavier than fertilized ones) were rarely detected in McMaster chambers, even when a high numbers of eggs was being excreted. For both methods there was a considerable variation in sensitivity between the different trials. This variation was largely explained by intensity of egg excretion (FEC) and factors inherent to the different laboratories involved in the trials and the countries where they were located. The probability of the diagnosis of STH infections increased as the number of eggs excreted increased. Although this finding is not unexpected, it highlights the importance of quantifying infection intensity in future studies comparing diagnostic methods. This will enable ready comparison of the sensitivity reported in different studies.

The differences between countries/laboratories are not easily explained and are likely multi-factorial. An important factor, which may have contributed to this difference, is human error. Although we employed standardized methods throughout based on identical written protocols, small differences in processing samples and/or examination of the slides between laboratories/countries cannot be ruled out. This is particularly the case in the use of the Kato-Katz, for which the time between processing and examination is extremely difficult to standardize (in the present study ranging from 30 to 60 min), yet crucial for the detection of hookworm eggs [12]. Similar major inter-laboratory differences also became apparent when their performance of diagnostic testing for STH was compared between European and African laboratories [11]. Therefore in future, rigorous quality control for similar studies is recommended to minimize human error. A set of control samples from the same source could have been examined independently by the different laboratories involved (so-called ring test). However, this would have required preservation of the samples, which may itself have thwarted the interpretation of the quality control, and dispatch to the laboratories involved would have resulted in different time periods between collection of sample from the donor and fixation, and eventual assessment of FEC, adding yet more variables and uncertainties to the outcome. Preservation (e.g., formaldehyde) is known to alter the morphology/density of eggs, resulting in false negative test results and an underestimation of FEC [29]. Moreover, when preserved by the addition of a preservant in a liquid formulation, it would no longer be possible to process samples as fresh samples, as normally done under field conditions, because then centrifugation would have to be implemented to discard the preservant prior to assay. This additional step, therefore, is likely not only to generate extra variation in the test results, but also to concentrate the eggs, hence increasing the sensitivity and FEC [30]. Other factors which cannot be excluded are differences in fecundity of worms [31], the number of samples containing unfertilized eggs (*A. lumbricoides*), the diet of subjects or the proportion of *N. americanus*/*A. duodenale*. The diet varied considerably across the five participating countries, and thus differences in the quality of food consumed would have created differences in fat and roughage content, which may have influenced the buoyancy of helminth eggs, particularly for the McMaster method as it is based on flotation of the eggs. Our study did not distinguish between *N. americanus* and *A. duodenale* eggs, yet it was remarkable that the effect of magnitude of FEC on sensitivity differed markedly between countries only for hookworm (interaction term), suggesting that sensitivity may also vary between hookworms species. At present, it remains unclear which factor(s) is (are) causing the observed variation across laboratories/countries, however, differences in sensitivity between countries for the McMaster were less pronounced compared to Kato-Katz, indicating that the McMaster is a more robust method under field conditions.

The quantitative comparison revealed an overall positive correlation. Yet, the Kato-Katz method resulted in significantly higher FECs than the McMaster method for *A. lumbricoides*, but not for *T. trichiura* or hookworm. These findings partially confirm previous studies summarized by Knopp et al. (2009) [32], where differences in FEC between Kato-Katz and FLOTAC (a derivative of the McMaster method) were more pronounced for *A. lumbricoides* and hookworm, than for *T. trichiura*. It is clear that intrinsic aspects of both methods explaining the discrepancy in sensitivity for STH will also contribute to the discrepancy in FEC. In addition, it is important to bear in mind that the Kato-Katz method does not include the homogenization of a large mass of the stool sample (41.7 mg compared to 2 g for the McMaster) prior to examination, that in certain cases may result in higher counts, as eggs are not equally distributed among the sample [33], [34]. The level of quantitative agreement was not consistent across the different trials involved, but this can be explained mostly either by a small number of samples containing STH (type error II) or differences in sensitivity.

The present study also confirms that the use of a fixed multiplication factor of 24 for the Kato-Katz should be revised to enable more accurate quantification of the eggs excreted [16]. Although the mean of the multiplication factor adjusted for the mass of feces examined (23.7) approached the conventially used 24, there was considerable variation in the multiplication factor across the different samples ranging from 11 to 100. Moreover, FECs based on the fixed multiplication factor resulted in significantly higher FECs compared to those based on a multiplication factor adjusted for the actual mass of feces examined, which may explain the above described difference in FEC between McMaster and Kato-Katz.

The statistical simulation revealed that both methods provide reliable estimates of drug efficacies, supporting the use of both methods for monitoring large-scale treatment programs implemented for the control of STH in public health. However, the McMaster method has several advantages when a large number of samples need to be examined because the microscopy is readily performed, and all parasites can be examined simultaneously, in contrast to the Kato-Katz method where different clearing times for the different STH require re-examination at times optimal for different species [15]. These findings also confirms that FECR is preferred as a summary measure for assessment of drug efficacy, since it allows an accurate and realistic comparison of FECR across laboratories or the locations where the trials have been conducted, and this regardless of differences in sensitivity between trials.

In conclusion, this multinational study highlights considerable variation in the performance of two methods used for the diagnosis of STH, particularly for the commonly used Kato-Katz. Both the McMaster and the Kato-Katz methods are valid methods for monitoring large-scale treatment administration programs. Yet, the McMaster method seems more suitable for further standardization because of its robust multiplication factor, and allowing for simultaneous detection of all species of STH.

## Supporting Information

Checklist S1 **STARD checklist**
(DOC)

Table S1 **A detailed overview of the calculations made to assess the accuracy of estimating drug efficacy**
(XLS)
